# Berberine, a Herbal Metabolite in the Metabolic Syndrome: The Risk Factors, Course, and Consequences of the Disease

**DOI:** 10.3390/molecules27041351

**Published:** 2022-02-17

**Authors:** Anna Och, Marek Och, Renata Nowak, Dominika Podgórska, Rafał Podgórski

**Affiliations:** 1 Chair and Department of Pharmaceutical Botany, Medical University of Lublin, 1 Chodźki St., 20-093 Lublin, Poland; renatanowak@umlub.pl; 2 Parexel Poland Sp. z o.o., ul. Żwirki i Wigury 18a, 02-092 Warsaw, Poland; marek.och@parexel.com; 3 Department of Internal Diseases, Institute of Medical Sciences, Medical College of Rzeszow University, 35-959 Rzeszow, Poland; dpodgorska@ur.edu.pl; 4 Department of Biochemistry, Institute of Medical Sciences, Medical College of Rzeszow University, 35-959 Rzeszow, Poland; rpodgorski@ur.edu.pl

**Keywords:** berberine, metabolic syndrome, cancer, cancer prevention, obesity, type 2 diabetes mellitus, ischemic heart disease, stroke, myocardial infarction, atherosclerosis, polycystic ovary syndrome, clinical trials

## Abstract

In recent years, the health of patients exposed to the consequences of the metabolic syndrome still requires the search for new solutions, and plant nutraceuticals are currently being intensively investigated. Berberine is a plant alkaloid possessing scientifically determined mechanisms of the prevention of the development of atherosclerosis, type 2 diabetes, and obesity, as well as cardiovascular complications and cancer. It positively contributes to elevated levels of fasting, postprandial blood glucose, and glycosylated hemoglobin, while decreasing insulin resistance. It stimulates glycolysis, improving insulin secretion, and inhibits gluconeogenesis and adipogenesis in the liver; by reducing insulin resistance, berberine also improves ovulation. The anti-obesity action of berberine has been also well-documented. Berberine acts as an anti-sclerotic, lowering the LDL and testosterone levels. The alkaloid exhibits an anti-inflammatory property by stalling the expression of cyclooxygenase 2 (COX-2) and prostaglandin E2. Berberine is neuroprotective and acts as an antidepressive. However, the outcomes in psychiatric patients are nonspecific, as it has been shown that berberine improves metabolic parameters in schizophrenic patients, acting as an adjuvant during antipsychotic treatment. Berberine acts as an anticancer option by inducing apoptosis, the cell cycle arrest, influencing MAPK (mitogen-activated protein kinase), and influencing transcription regulation. The inhibition of carcinogenesis is also combined with lipid metabolism.

## 1. Introduction

**Figure 1:**
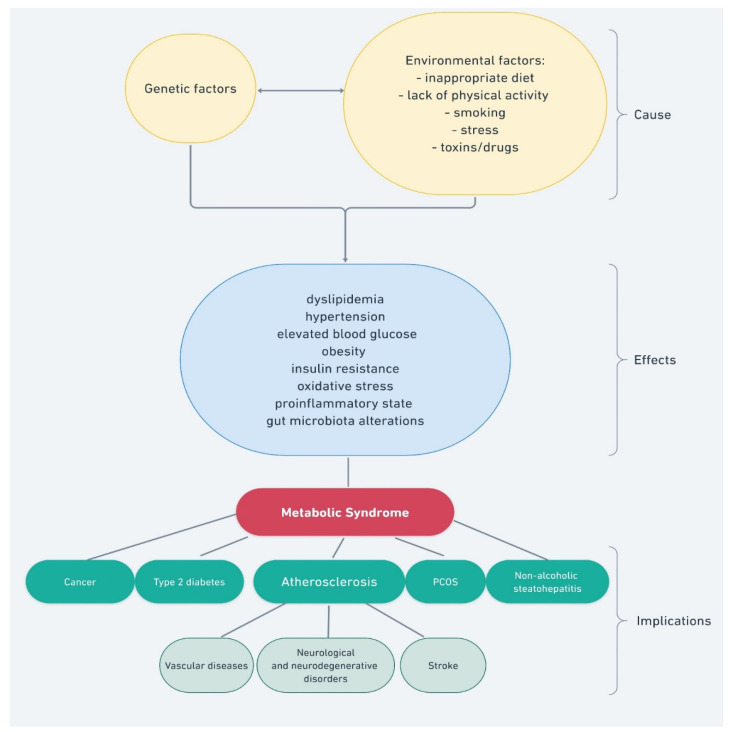
Factors and processes that impact the development of the metabolic syndrome.

The metabolic syndrome, also called syndrome X, is a set of interrelated factors that significantly increase the risk of developing atherosclerosis and type 2 diabetes (T2DM) and, consequently, cardiovascular disorders [1]. The risk of developing the metabolic syndrome, and its course severity, can be reduced by eliminating risk factors, such as an inappropriate diet, physical inactivity, stress, anxiety-depressive disorders, personality disorders, and the resulting behavioral addictions (Figure 1).

The fight against this complex disease also involves the treatment of identified disorders within the metabolic syndrome, such as atherogenic dyslipidemia (hypertriglyceridemia and lowering LDL cholesterol), as well as the treatment of the repercussions of the metabolic syndrome, such as atherosclerosis, diabetic feet, liver cirrhosis due to non-alcoholic or alcoholic steatohepatitis, acute or chronic pancreatitis, local or general immunodeficiencies, and tendency for recurrent infections [2,3,4]. A consequence of the metabolic syndrome is an increased risk of some malignant neoplasms. In the course of the metabolic syndrome, disorders of menstruation, ovulation, and fertility can also be observed, as well as miscarriages and those resulting from cerebral microcirculation disturbances, such as psychoorganic and mood disorders [5,6].

**Figure 2:**
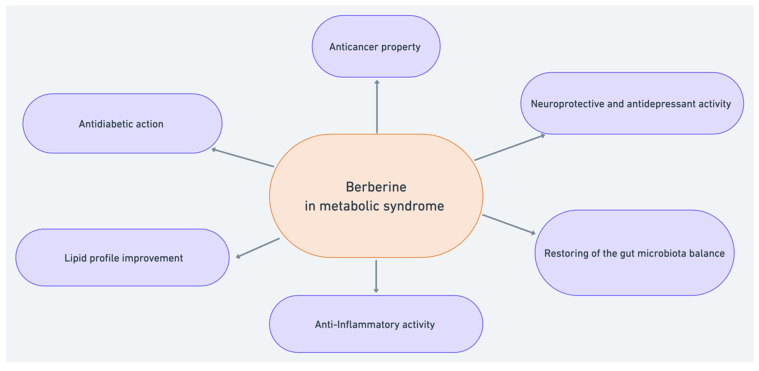
Potential berberine activity against the metabolic syndrome.

Berberine is an alkaloid with strong pharmacological activities that are currently receiving great interest. Berberine has always been used in traditional medicine as a plant extract, but new research methods have established that berberine is a promising treatment for current diseases. A recent study has confirmed the significance of its anticancer activity and its effectiveness in neurological, metabolic, and cardiovascular disorders. The compound has been subjected to multiple clinical evaluations in patients with the metabolic syndrome, and its use in related diseases (Table 1) [7]. The graphic summary of the potential action of berberine in the risk, course, and consequences of the metabolic syndrome is presented in Figure 2.

The authors searched the following databases: PubMed, Scopus, Springer, Taylor & Francis Online, and Google Scholar using the keywords: berberine and: metabolic syndrome, inflammation, cholesterol level disorders, obesity, diabetes mellitus, depression, mental disorders, cardiovascular diseases in recent 30 years. A review of more than 2000 studies on the metabolic syndrome and the biological activities of berberine, and its in vitro and in vivo properties, was carried out, and 173 manuscripts were selected regarding the description of the actions and the potential use of the alkaloid in patients at risk of a sequelae of syndrome X disorders.

## 2. Antidiabetic Action of Berberine 

The metabolic syndrome is, among others, defined as a cluster of glucose intolerance, central obesity, and insulin resistance as the source of pathogenesis. Today, the fight against insulin resistance and obesity concerns a growing group of ever-younger patients. It has also become a key point in the fight against infertility. Berberine antidiabetic properties in T2DM were first documented in 1986 [20] and its antidiabetic activity has been proven in vivo [21,22,23]. Its antidiabetic activity is the best-studied potential therapeutic application of berberine. Unfortunately, despite its beneficial effects and high safety profile, its poor bioavailability is still a limitation in its clinical application. 

Previous research showed that berberine mitigates insulin resistance [24], and the reduction in alanine and aspartate transaminase levels, in patients with T2DM [20]. Furthermore, it was shown that berberine positively contributed to elevated levels of fasting and postprandial blood glucose and glycosylated hemoglobin, while decreasing insulin resistance. 

Berberine stimulates glycolysis by increasing the activity of glucokinases, improving insulin secretion, and inhibiting gluconeogenesis and adipogenesis in the liver [25,26,27]. By the activation of 5-adenosine monophosphate kinase (AMPK), it improves insulin sensitivity in individuals with insulin resistance and increases the translocation of the glucose-4 transporter into the plasma. It has also been reported that, in individuals with insulin resistance, where the signaling pathway of protein kinase B (Akt) is impaired, berberine increases Akt phosphorylation and, thus, activates Akt, precisely by the activation of AMPK. According to scientific reports, the pathways through which berberine regulates glucose uptake may be diverse, but the activation of the AMPK pathway is the most likely. Berberine also enhances the expression of the AMPK-dependent adipose tissue triglyceride lipase, which is positively associated with long-term weight loss and is one of the mechanisms of action in the prevention of obesity [20].

Berberine has also been shown to increase glucose-stimulated insulin secretion [25]. As has been shown both in vitro and in vivo, the compound increases insulin secretion in islet cells by increasing the level of glucagon-like peptide-1 (GLP-1) [28]. As berberine inhibits glycosidase, it may also reduce glucose transport across the intestinal epithelium. Thus, it may exert an antihyperglycemic effect [29]. However, it is poorly absorbed after oral administration, and only nanomolar plasma concentrations can be achieved both in humans and animals [30]. Due to its high therapeutic potential, it is compared to metformin. Simultaneously, the high efficacy and safety profile gives berberine some advantages in its use, e.g., in patients who do not tolerate metformin therapy. Not only does berberine appear to produce significantly better results than metformin in blood glucose regulation, it also supersedes the benefits of rosiglitazone by improving fasting blood glucose levels. Although further research on the efficacy of berberine administered to patients with T2DM is needed [31], the prospects of combining oral hypoglycemic medications with berberine result in positive outcomes. Similar to metformin, berberine controls several effectors, such as mitogen-activated protein kinase [32].

The mechanism of berberine efficacy in obesity is also being currently intensively investigated, especially in cases connected with T2DM. The influence of berberine on insulin resistance was clinically evaluated in obese women with polycystic ovary syndrome, and improved insulin resistance was indicated [33,34]. In obese patients with cardiometabolic syndrome risk factors [35], the improvement of lipid parameters (total cholesterol, LDL, TG, the cholesterol/HDL ratio, and the TG/HDL ratio), weight, and fat mass loss were observed. In obese patients, c-Jun N-terminal kinases (JNK) [36] and excitotoxic neurotransmitters are involved in the development of insulin resistance in ischemic conditions [37], and berberine affects the lipid metabolic pathways mediated by those kinases, as well as acting as a neuroprotectant. This activity also translates into its anticancer properties, as discussed below [38,39,40,41]. Additionally, the influence on body mass has been found in several mechanisms that were confirmed in humans, and it is partially connected with its antidiabetic actions. In vitro studies have shown that berberine significantly decreased the amount and size of lipid droplets found in the 3t3-L1 adipocyte cell lines. Berberine has been clinically shown to inhibit α-glycosidase [32]. There was also a clinically proven inhibitory effect on adipocyte differentiation by reducing the expression of liver X receptor alpha, the peroxisome proliferator-activated receptor (PPARγ), and the sterol element-binding protein-1 receptor (SREBP). Berberine also enhances basal triglyceride lipolysis in adipocytes, and inhibits the separation and augmentation of preadipocytes and adipocytes through the PPARγ and CCAAT/enhancer-binding protein α pathways [20]. These mechanisms underlie the understanding of the effects of berberine on the treatment and prevention of obesity.

Regarding polycystic ovary syndrome, berberine’s efficacy is currently being investigated and the outcomes are important for the treatment of patients with this condition. Research suggests the high potential of berberine administration in patients with polycystic ovarian syndrome, as it reduces insulin resistance and improves ovulation [42]. Three months of 500 mg of berberine treatment showed a significant improvement in the lipid profile 3 in 26 patients with polycystic ovary syndrome, as well as increasing the pregnancy rate and decreasing the appearance of severe ovarian hyperstimulation syndrome [43]. Substituting metformin with berberine also resulted in a decrease in the negative effects, lower lipid parameters, and body mass index (BMI) in such patients [24]. Further research showed that berberine, combined with Yasmin, has been described to significantly improve sex hormones and glucose metabolism [20].

In addition, berberine, combined with letrozole, has a synergistic effect on ovulation induction in polycystic ovary syndrome with insulin resistance. The effect is better than metformin combined with letrozole. Berberine can significantly improve ovulation rates [44]. On the other hand, another study [45] revealed that letrozole, combined with berberine, did not improve fertility in patients with polycystic ovary syndrome, to some extent [46].

Currently, researchers pay high attention to the role of nutraceuticals in T2DM, and some of them are being intensively investigated. Some nutraceuticals have been reported to decrease postprandial and fasting glucose levels in plasma, glycated hemoglobin, and fasting plasma insulin. Several clinical trials and further research indicated that berberine, as a nutraceutical, amalgamated with chromium picolinate, inositol, curcumin, and banaba, was positively associated with the reduction in inflammation in patients with dysglycemia, as well as the improvement in glycometabolic compensation, triglycerides, and cholesterol values. Research has shown that the level of the high-sensitivity C-reactive protein (CRP) in patients with fasting dysglycemia decreased after 3 months of combined nutraceutical treatment [47]. The combination of berberine and *Bifidobacteria* administered to patients with pre-diabetes and diabetes mellitus also indicated their supportive roles in diabetes treatment [15].

## 3. The Cholesterol-Lowering Effect of Berberine 

An abnormal lipid profile characterizes patients with the metabolic syndrome. It is a disease component, or risk factor, for the appearance of the metabolic syndrome, and is characterized by elevated serum triglycerides, LDL, and low HDL cholesterol levels. Although the causes of lipid metabolism disorders can be complex, nutrition modification is one of the key avenues for the prevention and treatment of cholesterol-related disorders that are associated with metabolic syndrome. Apart from basic nutritional changes, the use of nutraceuticals, or the supplementation of plant compounds with proven biological activity, such as extracts or individual plant compounds, appear to be useful today.

Berberine is one of the plant metabolites with a high interest nowadays, with a beneficial effect on the lipid profile, and the mechanism of its action in this area has already been partially ascertained. The primary mechanism for lowering cholesterol is the inhibition of intestinal absorption by interfering with the cholesterol micellization in the gut, and by reducing cholesterol absorption and secretion by enterocytes [48]. The compound also stimulates the accumulation of bile acids [49], regulates the excretion of cholesterol, and stimulates the removal of LDL cholesterol from the blood [50,51]. Molecularly, as described above, berberine stimulates the AMPK responsible for fatty acid synthesis [32]. Berberine has also been shown to regulate liver cholesterol biosynthesis through the increased phosphorylation of 3-hydroxy-3-methylglutaryl coenzyme A reductase, which catalyzes the rate-limiting step in cholesterol biosynthesis [52].

Clinical trials have confirmed the antisclerotic activity of berberine. Elevated levels of HDL, TG, and LDL, and decreased levels of TG, have been demonstrated in patients after 3 months of berberine treatment [53]. Significant reductions in blood glucose and insulin, after standard mixed meals, and increased flow-mediated dilation and a significant reduction in arterial systolic blood pressure were observed [10]. Recent clinical reports state that, in addition to the beneficial effect on the lipid profile, berberine increases the level of testosterone in men, which may reduce the risk of developing cardiovascular diseases. Importantly, previous studies have shown that berberine (1500 mg/day for 3 months) reduced testosterone in women with PCOS [8]. 

As described earlier, the nutraceutical combinations seem to also play a role in the modulation of the lipid profile, and they have been clinically assessed. Several berberine-containing nutraceutical mixtures have been evaluated in clinical trials and have shown promising effects in patients with an abnormal lipid profile. One of them, conducted in patients with a low-to-moderate risk of hypercholesterolemia, has revealed that the combinations of nutraceuticals containing berberine, policosanol, and red yeast rice extract reduces the total cholesterol and LDL after 4 weeks. There were no significant changes in HDL, fasting glucose, and the serum triglycerides concentrations in any of the study groups, and the complex was safe and well-tolerated [9]. In patients with low-grade systemic inflammation, the oral administration of red yeast rice, berberine, and policosanol improved the lipid profile and attenuated the degree of systemic inflammation and endothelial injury. A significant reduction in total cholesterol, LDL cholesterol, high-sensitivity CRP, and endothelial microparticles were also observed in these patients [11]. The combination of berberine, with fermented red rice and chitosan, significantly reduced non-HDL-C, LDL-C, and apolipoprotein B after 12 weeks of treatment, compared to the placebo. On the other hand, there were no changes that were observed between the treatment arms in HDL-C, triglycerides, fasting plasma glucose, glycated hemoglobin (HbA1C), the waist circumference, and BMI [12].

Clinical studies have also shown that, after 12 weeks of the administration of berberine, in combination with chlorogenic acid and tocotrienols, in menopausal women at risk of dyslipidemia, cholesterol and LDL levels were reduced. Further studies on the influence of berberine on the symptoms of menopause are necessary. Furthermore, the side effects of berberine are minimal, and mainly affect the digestive system [54].

Berberine was also assessed in combination with changes in lifestyle. Studies showed that in patients with non-alcoholic fatty liver disease, berberine, combined with lifestyle changes, contributed to a significant loss of liver fat, body weight, and an improved serum lipid profile, much more so than in patients who adapted only to a new lifestyle or the use berberine as supplements alone. In this study, berberine also weakened the efficacy of pioglitazone and indicated its regulatory capacity for lipid metabolism in the liver [55].

The effect of berberine, as well as its activity in combination with nutraceuticals, is undoubtedly beneficial in patients with a disturbed lipid profile. Consequently, it is interesting from the point of view of preventing the occurrence of cardiovascular diseases, because hypercholesterolemia is considered one of the most important cardiovascular risk factors.

## 4. Berberine and Gut Microbiota

Berberine may also reduce the risk of developing the metabolic syndrome through its beneficial effects on the gut microbiota. In the last decade, many studies have indicated that the composition of gut microbiota is associated with the regulation of the host’s health and metabolism. Dysbiosis, defined as an alteration in the quality and/or quantity of the intestinal microbiota, can affect the host’s physiology [56] and may be a factor that leads to the onset of various diseases, including obesity and T2DM [57,58,59], as well as cardiovascular diseases, Crohn’s disease, and cancer [60,61]. Obesity and T2DM are closely related to a low-grade inflammatory state with the abnormal expression and production of many inflammatory mediators, such as interleukins and tumor necrosis factors [48]. Understanding the molecular mechanism of the action of berberine is still poor, and due to its very low bioavailability after oral administration, it seems highly likely that it acts by affecting the composition of the gut microbiota. The impact on the host’s metabolic homoeostasis has been implicated in several microbial metabolic pathways that regulate the production or transport of amino acids, short-chain fatty acids (SCFA), or bile acids (BA) [62,63,64]. Recent studies have shown that obesity is related to a higher number of *Firmicute phylum* and a relatively lower number of the *phylum Bacteroidetes* [65,66], whereas another study indicated that the proportion of *Firmicute phylum* and the *Clostridia* class in the intestinal tracts of patients was relevantly reduced [67]. The disturbance in the *Firmicutes*/*Bacteroidetes* ratio, which are two major components of the gut microbiota, was observed in many pathological conditions. In obesity, the *Firmicutes/Bacteroidetes* ratio is shifted into a higher content of *Firmicutes*, and berberine administration at a dose of 150 mg/kg is able to restore the balance, by declining *Firmicutes* abundance and slightly increase in *Bacteroidetes* [68]. Another study has shown that berberine, administrated at a dose of 200 mg/kg for six weeks, relevantly diminished the relative abundances of *phylum Bacteroidetes* and *Firmicutes* in the gut of high-fat diet-fed mice, and its antimicrobial activities may result in a lower degradation of dietary polysaccharides, decreasing the potential calorie intake, and, subsequently, systemically increasing fasting-induced adipose factor gene expression in visceral adipose tissues [69]. 

The disturbance in the ratio of *Firmicutes*/*Bacteroidetes* in the gut microbiota could be associated with a variety of diseases, including obesity [70]. It has also been proven that berberine reduces the diversity of the gut microbiome and changes the relative abundance of *Eubacterium*, *Desulfovibrio,* and *Bacteroides* [71]. In the course of inflammatory diseases of the digestive system, such as inflammatory bowel disease or colitis, berberine can decrease the prevalence of harmful bacteria, such as *Enterococci* and *E. coli,* and can increase the total relative abundance of *Lactobacilli* and *Bifidobacteria* [72]. *Lactobacillus* sp., a member of *Firmicutes*, was found to be inhibited by berberine in vitro [73]. An experiment on mice (C57BL/6) has shown that berberine, at a dose of 300 mg/kg, reduced the populations of *Ruminococcus schinkii*, *Ruminococcus gnavu*, *Lactococcus lactis*, *Lactobacillus acidophilus*, and *Lactobacillus murinus,* and enriched the population of *Bacteroides* [74]. This study has also evaluated the impact of berberine on the profile of bile acids and the gut microbiota. Tian and colleagues have reported that the short-term intake of berberine at a dose of 100 mg/kg alters gut microbiota by lowering *Clostridium* clusters IV and XIVa, as well as their bile salt hydrolase activity, which leads to the accumulation of taurocholic acid. Taurocholic acid may activate the intestinal farnesoid X receptor (FXR), which can influence lipid, glucose, and bile acid metabolism [75]. Many observational studies have indicated a connection between elevated levels of circulating branched-chain amino acids (BCAAs) and a poor metabolic condition. High BCAA blood levels are positively correlated with insulin resistance. Berberine has ability to reduce the relative abundance of BCAA-producing bacteria, including *Clostridiales*; the families of *Streptococcaceae*, *Clostridiaceae*, the *Streptococcus genera*; and *Prevotella* [76]. Berberine can also regulate the circulating levels of BCAAs and improve glycemic control in both healthy participants and patients with T2DM [77]. Berberine fumarate, an organic acid derivative of berberine, has a better oral bioavailability, reducing inflammation, inhibiting the overexpression of the toll-like receptors and phosphorylated c-Jun N-terminal kinases, increasing the expression of glucose transporter-2, phosphoinositide 3-kinase, and other proteins related to oxidative stress that lead to alleviated metabolic disorders and an improved control of glucose metabolism in T2DM [71]. 

It has been also reported that berberine affects the bacteria that produce short-chain fatty acids and bile acids in the gut microbiome [78,79]. Bile acids regulate blood levels of cholesterol, triglycerides, glucose, and energy homeostasis. This may prove that berberine acts in a similar manner to some anti-diabetic drugs, such as acarbose and metformin, by modifying the gut microbiota and, thereby, altering the composition of bile acid and increasing the ratio between primary and secondary bile acids, which evince proinflammatory and cytotoxic effects [71,80,81]. A recent study revealed that berberine, at a dose of 4 g per day, administered orally for 12 weeks, lowered the gut species that mainly produce SCFA or single sugars from digested polysaccharides or oligosaccharides, including *Ruminococcus bromii, Faecalibacterium prausnitzii, Bifidobacterium* spp., and *Roseburia* spp., as well as inversing two *Bacteroides* spp. and multiple taxa of *γ-Proteobacteria*. [82]. These results showed that berberine reduced the transformation of microbial bile acids, especially the production of deoxycholic acid by *Ruminococcus bromii* and, therefore, decreased FXR activity, which may explain its antidiabetic effect. In the liver, bile acids activate FXR, which serves as a suppressor of BA synthesis and promotes the enterohepatic circulation of bile acids. FXR induces the expression of a small heterodimer partner that suppresses the liver receptor homolog-1, leading to the lowered transcription of bile acid-synthetic enzymes-Cyp7a1 [83]. Guo and colleagues have reported that, in the livers of mice administered orally high doses of berberine (300 mg/kg), the expression of Cyp7a1 and Cyp8b1, and an uptake transporter, sodium taurocholate, in its co-transportation of polypeptides, were significantly increased [74]. Research has shown that some of the effects of berberine could be related to the increase in the population of SCFA-producing bacteria, which has a relevant influence on blood glucose and lipid levels [79,84]. SCFA, such as butyric acid, acetic acid, propionic acid, isobutyric acid, isovaleric acid, and valeric acid can mitigate the inflammation of the bowel mucosa and can enter the bloodstream, reducing lipid and glucose levels [85]. It has been shown that the treatment of high-fat diet-induced obese animals with berberine (100 mg/kg and 200 mg/kg) and metformin induced an increase in the total relative abundance in seven operational taxonomic units (OTUs) from less than 2% to 10–20%. Six of these OTUs were major SCFA-producing bacteria, such as *Bacteriodes*, *Blautia*, *Butyricoccus,* and *Phascolarctobacterium* [86]. Zhang et al. using the mouse model of obesity, diabetes, and dyslipidemia, showed that berberine, at a dose of 136.5 mg/kg, reduced body weight, food intake, blood glucose, and HbA1c levels, among other associated increases in the numbers of SCFA-producing bacteria (*Butyricimonas*, *Ruminococcus*, and *Coprococcus*), as well as reducing the population of opportunistic pathogens (*Prevotella* and *Proteus*) [87]. The upregulation of GLP-1 and peptide YY, induced by butyrate, can be relevant for the prevention and treatment of insulin resistance and obesity [88].

Most of obtained data derived from animal models, and further studies on human are required to assess these findings because they do not relate mutually. We still do not have detailed knowledge on how the host’s gut microbiota responds to berberine intake and to what extent changes in gut flora composition are related to the metabolic benefits of berberine, including its anti-obesity and anti-diabetic effects. Qin and colleagues carried out a two-stage metagenome-wide association study using deep shotgun sequencing of fecal samples to find changes in gut microbiota in a group of 345 patients with T2DM. They identified T2DM-related gut flora dysregulation, which was associated with an increase in the opportunistic species of pathogenic bacteria and a decrease in butyrate-producing bacteria, confirming previous findings in animal models [89]. 

## 5. Anti-Inflammatory Activity of Berberine

One of the conditions that make up the metabolic syndrome is the occurrence of chronic inflammations. They are a serious health problem for patients affected by syndrome X. There is a consensus that inflammatory pathways contribute to the pathogenesis of the metabolic syndrome, and new research indicates that inflammation plays a key role in the development and progression of the metabolic syndrome. However, up to today, the specific pathways associated with this disease are scantily understood. Monocyte-derived chemokines and cytokines promote inflammation and insulin resistance. Inflammatory biomarkers (CRP, fibrinogen, and serum amyloid A), cytokines, and chemokines have been associated with the pathogenesis of the metabolic syndrome. The treatment of existing inflammations using glucocorticosteroids also promotes the progression of the metabolic syndrome.

On the other hand, inflammations can also occur as a result of disorders observed over the course of the disease. In the case of obesity, adipose tissue increases the occurrence of inflammations by releasing pro-inflammatory adipokines (leptin and chemerine) and disrupting the anti-inflammatory activity of adiponectin. Most cells in the subcutaneous adipose tissue promote both inflammation and fibrosis. Inflammations may also appear as a distinct disease entity within the metabolic syndrome, such as osteoarthritis, back pain syndromes, and acute or chronic pancreatitis [90,91,92,93,94,95].

The anti-inflammatory effect of berberine has been known for several years, but it has not yet been fully understood. The compound exhibits an anti-inflammatory property both in vitro and in vivo. It curbs interleukin (IL)-1, IL-6, and tumor necrosis factor (TNF)-gene transcription by diminishing levels of inflammatory proteins. It further stalls the expression of cyclooxygenase 2 (COX-2) and prostaglandin E2 [96], it halts the NF-κb signaling pathway, it inhibits IL-8 production in cancer cells [97], and it hinders the increase in NO and TNF-α [96,98]. Berberine has been found to decrease the COX-2 transcriptional activity observed in colorectal cancer cells [99]. In the near future, we can expect the results of clinical trials of the effects of berberine on the levels of CRP, IL-1β, IL-6, and TNF-α [23]. The anti-inflammatory activity of berberine makes it noteworthy as a supplement or nutraceutical due to its potential to prevent the development of colorectal cancer [99].

## 6. Anticancer Activity of Berberine 

An implication of the metabolic syndrome is the increased risk of some malignancies. The most common are colon cancer, esophageal adenocarcinoma, postmenopausal breast cancer, endometrial cancer, kidney cancer, non-small cell lung cancer in smokers, and liver cancer in patients with cirrhosis.

The anticancer properties of berberine are currently being studied in a very intensive way, and the results make it even more noteworthy as a potential nutraceutical for patients suffering from syndrome X that are at risk of developing cancer. Due to the pharma-codynamic limitations of the compound, these studies have been performed mainly in an in vitro model and the molecular mechanism of the berberine anticancer activity has already been studied in detail. However, several clinical trials are currently being carried out on its antitumor activity. Currently, the first phase of the study is underway to prevent colorectal cancer in patients with ulcerative colitis in remission [23].

Berberine is cytotoxic to cancer cell lines and this activity depends on the dose and time. The therapeutic window of berberine, in most cases, is narrow and depends also on the type of cells that are treated [7]. For example, the cell viability of leukemic cell lines obtained by Och et al. was in the rage of 80 μM–250 μM, with 80 μM to CCRF/CEM; 80,15 μM to J45.01; 90,45 μM to HL-60; 110,05 μM to HL-60/MX1; 225,15 μM to CEM/C1; and 240,45 μM to U266B1. The HL-60/MX2 cells exposed to berberine did not fall below 50%, despite their exposure to the maximum concentrations possible that could be obtained in the in vitro experiment (250 μM) and in this case, the dose of IC50 could not be determined for this line due to the poor cytotoxicity of the compound [100]. 

Berberine results in the downregulation of the 33 genes involved in the cell cycle and cell differentiation. It is time- and dose-dependent [101]. Berberine arrests human cancer cells in the G1 phase in low concentrations. At high concentrations, it arrests the cell cycle in the G2/M phase [96,102]. Berberine inhibits the cell cycle in the G1 phase by the up-regulation of the B-cell translocation gene 2. This proliferation regulatory gene is induced by the p53 protein. The cell cycle arrest in the G2/M phase by berberine is p53-independent [102,103,104]. The phase arrest G0/G1 was reported in bladder cancer cell lines BIU-87 and T24, and the lymphocytic leukemia cell line L1210, after its exposure to berberine [105,106]. The colon cancer cells’ exposure to berberine from caused a phase cell cycle arrest of G0/G1 with the down-regulation of the anti-apoptotic gene BCL2 and was concentration-dependent [107,108,109]. One of the targets for berberine-induced cell cycle arrest is cyclins. Cyclin D1 was down-regulated after its exposure to berberine in the G1 cell cycle phase [102]. The lower expression of cyclin B1 and the increased expression of Wee1 can arrest tumor cells in the G1 and G2 phases after their exposure to berberine [102,110]. In MDA-MB-231 and MCF-7 breast cancer cells, after their exposure to berberine, G0/G1 arrest, which is possibly due to a decrease in the level of the cell cycle regulatory protein cyclin B1. This effect was dose dependent [102]. The cell cycle arrest in the G2/M phase by berberine is dependent on the REV3 gene [111]. The inhibition of the cell cycle in the G2/M phase after its exposure to berberine has also been described in colorectal cancer cells of the SW480 line [112].

Apoptosis is one of the most extensively researched and documented berberine-induced processes. The proapoptotic properties of berberine have been confirmed after its exposure to alkaloids and the induction of biochemical events, such as a decrease in mitochondrial membrane potency, caspase activation, poly-(ADP-ribose)-polymerase (PARP) breakdown, or the release of cytochrome C or Bc12 proteins [113]. Berberine acts as a pro-apoptotic in tumor cells by its up-regulation of pro-apoptotic genes and its down-regulation of anti-apoptotic genes [97,100]. In leukemic cells exposed to berberine, changes in gene expression showed that even a low cytotoxic dose of berberine increased the expression of caspase genes CASP3, CASP8, and CASP9, as well as the pro-apoptotic genes BIK, BAX, and BAK1 with a simultaneous down-regulation of the expression of the anti-apoptotic genes BNIP1, BNIP3, BCL2, and BCL2L2 [100]. Moreover, in HL-60 [114], U937, and B16 lines [115], the activation of protein caspase-3 and -9, an increase in the Bcl2-associated X protein (Bax), and a decrease in the Bcl-2 protein level after its exposure to berberine were reported [114]. By investigating the pro-apoptotic activity of berberine, we can also find that other pro-apoptotic proteins are involved in the apoptosis signaling pathways, such as p53, retinoblastoma protein, caspase-8, Fas receptor (death receptor)/FasL (Fas ligand), ATM (serine/threonine kinase), BID (BH3 interacting domain death agonist, a pro-apoptotic member of the family of Bcl-2 proteins), and TNF. The levels of these proteins have been reported to increase after their exposure to berberine, while the levels of Bcl-X, Survivin (an antiapoptotic protein), c-IAP1 (an inhibitor of the apoptosis protein), and XIAP (the X-linked inhibitor of the apoptosis protein) decreased after their exposure to berberine. Berberine was also shown to regulate apoptotic proteins through an increase in the level of reactive oxygen species, one of the key apoptosis regulation agents [40,97,116]. 

The next target in berberine-induced apoptosis is the death receptors (DR), known as a tumor necrosis factor-related apoptosis-inducing ligand receptor (TRAIL). TRIAL has a great potential in cancer treatment. It induces apoptosis by binding to the aforementioned death receptors, i.e., DR4 and DR5, and it induces tumor cell death. TRAIL selectively induces apoptosis, and the development of resistance (partial or complete) limits its use. Berberine acts synergistically with TRAIL. Furthermore, it sensitizes cancer cells with resistance to TRAIL. In the TRAIL-sensitive MDA-MB-231 breast cancer cell line, and the TRAIL-resistant MDA-MB-468 human breast cancer cell line, berberine acts synergistically with TRAIL but it also sensitizes resistant cells, which was confirmed with the markers of the process: caspase-3, PARP 9 Poly (ADP-ribose) polymerase 1 cleavage, and p53. Moreover, in the 4T1 breast cancer cell line, despite its moderate cytotoxicity, berberine, in combination with antiDR5, inhibited the primary growth and reduced its metastasis to the lungs [110,117]. 

There are scientific reports on the effects of berberine on mitogen-activated kinases (MAP or MAPK) that are involved in the direction of cell responses. They regulate processes important in carcinogenesis, for example, apoptosis, mitosis, gene expression, proliferation, and differentiation [96]. Berberine modulates mitogen-activated protein kinase signaling pathways, such as the p38 MAPK extracellular signal-regulated kinase 1/2 (ERK1/2) and the JNK pathways.

The modulation of these pathways is noteworthy in the search for new potential anticancer drugs, and the effects depend on the cell type. Berberine activates MAPK in human colon cancer cells [118], human hepatoma cells (HepG2), and in non-small cell lung cancer cells [38,39]. In turn, in human HeLa cervical carcinoma cells, berberine enhances the phosphorylation of JNK and ERK1/2 but it inhibits the phosphorylation of p38 MAPK [119]. Furthermore, berberine reduces the phosphorylation of p38 MAPK, JNK, and ERK1/2 in gastric cancer cells [41]. This JNK/p38 MAPK signaling pathway is disrupted in many types of cancer [120]. Berberine was shown to suppress cancer cell invasion and migration in the gastric cancer SNU-1 cell line by blocking the JNK/p38 signaling pathway [40]. More precisely, berberine acts on MAPK through the impact of microRNA that inhibits the translation of certain proteins, whose dysfunction plays a role in the formation of cancer. The levels of these proteins are correlated with the tissue factor TF, which contributes to tumor metastasis and has been shown to activate signaling cascades, including MAPK. Apoptosis through the miR-19a/TF/MAPK signaling pathway has been described after exposure to berberine in human lung cancer A549 cells. Berberine lowers the level of TF and raises the level of miR-19a, thus activating MAPK signaling that leads to the apoptosis of cancer cells [39]. The cyclin-dependent kinase inhibitor p21 (CIP1/WAF1), which is involved in apoptosis, cell cycle control, DNA replication, and cell differentiation [52] is linked with the human protein Forkhead box O3 (FOXO3a) and p53 in control of cancer cell growth [121,122,123]. FOXO3a is a transcription factor that belongs to the family of transcription factors with tumor suppressor activities. It is regulated by the phosphatidylinositol 3-kinase/Akt signaling pathway, and its growth factor receptor-induced activation is connected to cell cycle arrest [124] and apoptosis [125] and, in general, with tumor suppression. It is known that the inhibition of FOXO3a causes tumor progression [126]. In non-small cell lung cancer, berberine induces apoptosis and inhibits proliferation by activating the p38α MAPK signaling pathway, resulting in increased levels of FOXO3a and p53 and the induction of the cell cycle inhibitor p21 (CIP1/WAF1) [38,126].

Berberine also acts against transcription factor 1 (AP-1). AP-1 is closely related to neoplastic transformation. It consists of complexes comprising of the following families of DNA-binding proteins: the Jun family (JunD, c-Jun, JunB, and v-Jun), the Fos family (Fra-1, c-Fos, FosB, and Fra-2), the Maf family (MafA, c-Maf, MafB, MafG/F/K, and Nrl) and the binding of ATF/cyclic AMP-responsive elements (b-ATF, ATF1-4, ATF-6, and ATFx), which play a key role in proliferation, apoptosis, and inflammation. AP-1 activity is regulated by UV radiation, infections, cell stress, growth factors, and cytokines [127]. Extrinsic carcinogens induce an increase in AP1 activity [128]. Many human tumors overexpress the Jun family [129,130,131]. This overexpression has been described in aggressive lymphomas [132,133] and in breast cancer [131]. On the other hand, the increased expression of c-Fos is described in endometrial cancer and osteosarcoma, while the decreased expression of c-Fos is observed in ovarian and gastric cancer [134]. AP-1 activation has been described to depend on the type of extrinsic stimulus and the cellular condition. For example, in the HepG2 line hepatoma cells after their exposure to berberine, the AP-1 protein was inhibited [99,135]. On the other hand, the inhibition of Lewis lung cancer metastasis from the mediastinal lymph nodes to the lung parenchyma was described through the activation of the AP-1 protein after the oral administration of berberine [136]. The oral administration of berberine also decreased the expression of the C-fos proto-oncogene [110]. In conclusion, the influence of berberine on the AP1 protein family is dependent on the cell type, and needs further investigation. 

In berberine, anticancer activity is also based on the influence on β-catenin. Mutations and the overexpression of β-catenin are associated with cancers, such as colorectal carcinoma, endometrial cancer, breast cancer, and ovarian tumors. In colon cancer cells, the expression of its mRNA is down-regulated by berberine. The alkaloid efficiently inhibits the nuclear level of β-catenin [137], regulates β-catenin negatively, and stimulates the expression of the adenomatous polyposis coli protein [96,110]. 

The metabolism of fats and lipids plays a role in the malignancies of the digestive system and is one of the advantageous mechanisms of the consequences of berberine in the metabolic syndrome. Berberine induces apoptosis in gastric cancer cells through the reduction of fatty acid accumulation and the reduction of FABP expression [138]. Berberine also down-regulates lipogenic enzymes, which are key in colon cancer. Berberine affects the SREBP-1 cleavage activating protein-1/sterol receptor element binding protein-1 pathway (SCAP/SREBP-1) that drives lipogenesis, inhibiting the pathway. As a result, the downregulation of lipogenic enzymes is observed, leading to the suppression of lipid synthesis linked to cell proliferation through the Wnt/β-catenin pathway [109]. Furthermore, the influence on JNK kinases plays a role in the anticancer and chemopreventive activity of berberine, in terms of the influence on lipid metabolism and its role in cancer development [139,140]. Current data from clinical trials indicate the chemopreventive potential of berberine in relation to neoplasms, such as colorectal cancer developing from adenomas and the prevention of adenoma.

Colon adenomas are precancerous lesions that develop into colon cancer. The removal of precancerous lesions is currently established to prevent colorectal cancer. Due to the high rate of recurrence of colorectal adenomas in patients after polypectomy, chemopreventive agents are sought to reduce the risk of the recurrence of colorectal adenomas. In patients with colorectal adenomas, after a complete polypectomy, receiving berberine twice daily was effective and safe. The risk of the recurrence of colorectal adenoma was reduced, making berberine an option for chemoprevention in patients after polypectomy [15]. 

Importantly, for oncological patients, berberine mitigates the effects of radiation therapy. In patients with lymphoma and cervical cancer, the mitigation of the effects of radiation therapy was described, and in patients with non-small cell lung cancer, berberine protected lung cells from damage induced by ionized radiation [141]. Berberine selectively sensitized tumor cells to ionizing radiation in patients with glioma [142]. 

## 7. Berberine in Mental Disorders within the Metabolic Syndrome 

As mentioned above, chronic stress, anxiety, depression, and personality disorders may lead to the development of the metabolic syndrome, and may be caused by it. Recent studies have shown that the prevalence of mental disorders, including severe conditions, such as schizophrenia, bipolar disorders, and depression is two to three times higher within the course of the metabolic syndrome, compared to the general population [143,144,145]. On the other hand, impaired glucose metabolism and dyslipidemia could have pathoplastic effects on psychiatric disorders [146]. Although certain antidiabetic drugs are helpful in controlling weight gain and elevated glucose levels during antipsychotic therapy, most conventional psychiatric drugs stimulate appetite receptors, which can lead to the development or progression of the metabolic syndrome [147]. Berberine administration in rats has been shown to significantly prevent olanzapine-induced weight gain and modulate the expression of many key genes that control energy expenditure. Berberine has also been shown to change the activity of biogenic amine neurotransmitters involved in the pathogenesis of the anti-psychotic drug-induced metabolic syndrome [148,149,150,151,152,153,154,155,156,157,158].

Researchers suggest that berberine acts as antidepressant; however, no clinical models have been conducted so far. Several studies in animal models suggested that berberine administration could help to promote optimal mental health by increasing the level of brain neurotransmitters, such as dopamine, serotonin, and norepinephrine, which are necessary to maintain proper brain functioning and a positive mood [52,159,160,161]. This effect results from the ability of berberine to inhibit monoamine oxidase activity, the main target of many antidepressant drugs [162,163]. Berberine, like other antidepressant drugs, affects sigma receptor 1. Studies also show that berberine can act as an antidepressant through the nuclear factor kappa-light-chain-enhancer of activated B-cells (NF-κB) signaling pathway, which is activated by oxidative stress. 

Recent findings reveal that the antidepressant effects of berberine result from the activation of the 5-HT2 receptor by its impact on the brain-derived neurotrophic factor-cAMP response element, the binding protein, and the eukaryotic elongation factor 2 pathways. These well-known antidepressant pathways are crucial for the antidepressant action of drugs. Berberine acts by increasing neurotrophic factor levels and restores the decreased levels of its mRNA [96,164].

Berberine has also been shown to be active in neurodegenerative disorders [165,166]. Recent studies have shown that it has a protective effect on the central nervous system [167,168] and that it exerts a neuroprotective effect by regulating the early immune activation of peripheral lymphocytes and immunotolerance in vivo [52]. However, this is not fully understood, and there are reports on berberine exacerbating neurodegeneration [159]. Furthermore, berberine significantly decreases kynurenine production, which, when increased, is metabolized to neurotoxic compounds (for example, quinolinic acid), and influences glutamatergic neurotransmission [96,164]. Berberine has also been described to inhibit the effects of rewards after the abuse of drugs, such as cocaine, morphine, and ethanol. It proceeds through the down-regulation of tyrosine hydroxylase expression or other mechanisms [165,169,170]. Berberine easily crosses the blood–brain barrier after its systemic administration, which increases its potential in the treatment of neurological diseases, but it needs further clinical investigation [165,166].

Current clinical trials of berberine in patients with schizophrenia are based on the hypothesis that berberine, as an adjuvant, can control weight gain and other metabolic symptoms associated with antipsychotic therapy. Studies are carried out to determine whether an adjuvant therapy with berberine limits weight gain in patients with schizophrenia who developed the metabolic syndrome [16,17,23]. 

## 8. Summary

Berberine is an alkaloid with strong pharmacological activities that are currently receiving great interest, and it is believed to be effective in patients with the metabolic syndrome in terms of risk factors, its course, and the consequences of the disease. Berberine prevents the development of atherosclerosis, DM2, and cardiovascular disorders. Furthermore, it has been shown to be administered preventively and it decreases the risk of developing the metabolic syndrome due to its neuroprotective and antidepressant activities. Berberine is also effective in the case of dyslipidemia, liver cirrhosis due to non-alcoholic or alcoholic steatohepatitis, infertility, and ovulation disorders. However, the most important and widely studied property of berberine is its anticancer activity, which is crucial, since the implication of the metabolic syndrome is a significantly increased risk of certain malignant neoplasms. Despite intensive research, there is still much confusion about the detailed effects of berberine. An example is the fact that the impact of berberine on certain pharmacological parameters is gender-specific, which requires further research. Zhao et al. suggests that berberine has different effects on testosterone in men than in women. This concerns women with PCOS, who tend to have higher testosterone, and concludes that the effects could be different in women with normal endocrine parameters. As such, a further examination of the effects of berberine on endocrine factors, such as sex hormone binding globulin, are needed. 

Currently, several clinical trials with metabolic syndrome are being conducted with regard to the risk factors, course, and consequences of the disease, with outcomes expected in the near future. The most intensively investigated are colorectal adenomas, the spectrum of schizophrenia, other psychotic disorders, prediabetes (impaired fasting glucose and impaired glucose tolerance), stable coronary artery disease, diabetes mellitus, chronic kidney disease, non-alcoholic steatohepatitis, hypertension, endothelial dysfunction, blood pressure, and chronic kidney disease (Table 2).

The new derivatives and formulations of berberine are a crucial challenge for scientists, as a low bioavailability and poor pharmacokinetic parameters of berberine still remain an obstacle in its potential usage. The development of new formulations and derivatives with similar biological activities, that are not limited by low pharmacological parameters, seem to be the most important targets. Compounds, based on berberine, that are effective at lower concentrations and have stronger biological activities are currently being intensively investigated. The latest articles provide information on the significant relationship between its structure and activity. New derivatives exhibit similar parameters of biological activity and are promising for further research.

## Figures and Tables

**Table 1:** Completed clinical trials with berberine in the metabolic syndrome based on clinicaltrials.gov (accessed on 10 Fabruary 2022).

Title	Conditions	Phase	Subjects	Duration	Interventions Given	Measures	Results in Berberine or Nutraceutical Combination Groups
A Mechanistic Randomized Controlled Trial on the Cardiovascular Effect of Berberine	Cardiovascular risk factor	Phase 2 Phase 3	84 men of Chinese ethnicity aged 20 to 65 years with hyperlipidemia, not currently receiving hormone replacement therapy such as testosterone replacement therapy in the past 12 months; not currently taking berberine or traditional Chinese medicine that contains berberine in the past 1 month; free of congenital diseases, infectious diseases, anemia, and glucose-6-phosphate dehydrogenase deficiency; and no history of any chronic diseases. including ischemic heart disease, myocardial infarction (heart attack), stroke, diabetes, cancer, liver/renal dysfunction, and gastrointestinal disorders	12 weeks	Berberine verus placebo	Lipid profile LDL-cholesterol, HDL-cholesterol Triglycerides and total cholesterol Blood pressure Systolic blood pressure and diastolic blood pressure in mmHg Thromboxane A2 Testosterone Body mass index (BMI) Waist-to-hip ratio Fasting glucose Fasting insulin Liver function: Alanine transaminase (ALT), aspartate aminotransferase (AST), alkaline phosphatase (ALP), total bilirubin, gamma-glutamyltransferase, total protein, and albumin Sex hormone binding globulin (SHBG) Thrombin time	Reduction in total cholesterol and HDL-C Berberine was safe with no serious adverse events Reduction in LDL-C Increase in testosterone in males No differences in triglycerides, thromboxane A2, blood pressure, BMI, or waist-to-hip ratio [8]
Long-term efficacy and tolerability of a nutraceutical combination (red yeast rice, policosanols, and berberine (MBP-NC)) in patients with low-moderate risk of hypercholesterolemia: a double-blind, placebo-controlled Study of the	Hypercholesterolemia	Phase 4	60 adults between 18 and 60 years with newly diagnosed primary hypercholesterolemia, not previously treated, after a run-in period of 3 weeks on a stable hypolipidic diet, with a body mass index between 18,5 ad 29,9 Kg/m^2^, serum low-density lipoprotein cholesterol above 150 mg/dL, and an estimated 10-year cardiovascular risk of <20% according to the Framingham risk scoring	Assessement after 4, 12 and 24 weeks of treatment.	Nutraceutical combination of red yeast rice extract (monacolins), berberine, and policosanols after dinner, in addition to the hypolipidic diet versus placebo	Level of cholesterol Level of tryglicerides	A significant reduction in total cholesterol and LDL-C at week 4 No significant changes in the concentrations of HDL-C, fasting glucose, and serum triglycerides in any of the groups MBP-NC was safe and well-tolerated [9]
effects of Armolipid Plus on cholesterol levels and endothelial function (mixture of berberine, policosanol, and red yeast)	Hyperlipidemia Endothelial dysfunction	Not Applicable	50 adults aged between 18 and 70 with total cholesterol levels > 220 mg/dL; LDL-cholesterol > 130 mg/dL; and with concomitant pathology, such as diabetes, chronic heart failure, coronary artery disease, arterial hypertension, and dysthyroidism, if stable in the previous three months	6 weeks	Mixture of berberine, policosanol, red yeast versus placebo	Percentage change from baseline of total cholesterol, LDL-cholesterol (LDL-C), HDL-cholesterol (HDL-C) and Triglycerides (Tg) plasma concentrations Improvement of endothelial dysfunction	Significant reduction in the homeostasis model assessment of insulin resistance (HOMA-IR index) Significant decrease in total and low-density lipoprotein cholesterol Triglycerides, high density lipoprotein cholesterol, and oral glucose tolerance test (OGTT) were not affected Significant reductions in blood glucose and insulin after the standard mixed meal Increase in flow-mediated dilation (FMD) and a significant reduction in arterial systolic blood pressure [10]
Nutraceutical combination in patients with low-grade systemic inflammation (berberine 200 mg, monacolin K 3 mg, chitosan 10 mg, and coenzyme Q 10 mg)	Atherosclerosis inflammation Hypercholesterolemia	Phase 4	100 adults aged 25 to 75 with suboptimal LDL cholesterol levels (LDL 100–160 mg/dL) and hsCRP levels of >2 mg/L, randomized after 30 days of a low-cholesterol diet	3 months	Nutraceutical combination: red yeast rice extract (monacolins), policosanol, berberine, folic acid, coenzyme Q10, and astaxanthin with a low-cholesterol/low-saturated fat diet and a regular aerobic physical activity schedule versus a low-cholesterol/low-saturated fat diet and a regular aerobic physical activity schedule + placebo	Change in LDL cholesterol Change from baseline in circulation endothelial microparticles Change in CRP	Significant reduction in total and LDL cholesterol hsCRP significantly reduced LDL cholesterol change was positively associated with hsCRP and EMP changes hsCRP and EMP changes were associated with each other [11]
The efficacy and tolerability of coleosoma nutraceutical formulations in dyslipidemic subjects (fermented red rice, berberine, and chitosan)	Dyslipidemias	Phase 2	39 adults aged 18 to 75 with non-HDL cholesterol ≥ 160 mg/dL	12 weeks	Coleosoma-patented dietary supplement composed of berberin, fermented red rice from monascus purpureus (monacolin K), chitosan, and coenzyme Q10	Change in non-HDL Change in non-HDL cholesterol Change in free plasma glucose Change in body mass index Change in waist-to-hip ratio Change in HbA1C (%) Difference in the HbA1C value Change in LDL cholesterol, triglycerides, and HDL cholesterol Difference in the LDL Cholesterol, triglycerides, and HDL cholesterol Change in ApoB/Apo A1 ratio Difference in the ApoB/Apo A1 ratio Change in inflammatory cytokines (IL-1, IL6, IL-10, hsPCR, and TNF-α) Difference in the inflammatory cytokine values Change in insulin Difference in the insulin value Change in the hormone profile (glucagon, active GLP-1, and GIP) Change in endothelial progenitor cells	Significantly reduced non-HDL-C Non-HDL-C significantly decreased Significant correlation between baseline level of non-HDL-C and the reduction observed after 12 weeks of treatment Significant reduction in LDL-C and apolipoprotein (Apo) B No changes were observed between treatment arms in HDL-C, triglycerides, fasting plasma glucose (FPG), glycated hemoglobin (HbA1C), waist circumference, and body mass index Differences in ApoB/ApoA ratio did not reach statistical significance [12]
Combined effects of bioactive compounds on the lipid profile (red yeast rice and policosanol composed of berberine, folic acid, and coenzyme Q10 (Armolipid Plus ^®^, Rottapharm))	Hyperlipidemia Low-density-lipoprotein-type Elevated triglycerides	Phase 2 Phase 3	118 adults with LDL-C plasma levels ≥ 130 mg/dL and ≤189 mg/dL that did not require lipid-lowering drug treatment according to the ATPIII guidelines, as well as adults that did not have cardiovascular disease, stroke, intermittent claudication, diabetes mellitus, renal issues, or effects/contraindications to lipid-lowering drug therapy	12 weeks	Armolipid Plus (red yeast, astaxanthin, berberine, policosanol, coenzyme Q10, and folic acid) versus placebo	LDL-C levels Cardiovascular risk Criteria for Metabolic Syndrome Levels of triglycerides and cholesterol high-density lipoprotein (HDL-C)	Plasma LDL-C reduced TC was reduced ApoB-100 was reduced The ratios of TC/HDL-C were reduced The ratios of LDL-C/HDL-C were reduced The ratios of ApoB-100/ApoA-1 were reduced No statistically significant changes were observed in TG and HDL-C levels The body mass index was reduced The weight loss observed in the AP consumption group had no significant impact on LDL-C reduction, Apo B-100, TC/HDL-C ratio, or on the ApoB/ApoA1 ratio Non-significant contribution of weight-loss to LDL-C reduction [13]
Effects of nutraceutical therapies on endothelial function, platelet accumulation, and coronary flow reserve	Hypercholesterolemia Endothelial dysfunction	Not Applicable	Adults aged between 18 and 70 years with hypercholesterolemia that did not require statins or were statin-intolerant	8 weeks	Combination A (Armolipid Plus): policosanol, red yeast rice (monacolin K), berberine, astaxantine, folic acid and coenzyme Q10 or combination B: berberine, red yeast rice powder (monacolin K), and leaf extract of *Morus alba*	Effects on endothelial function Evaluation of treatment tolerability Reasons for treatment discontinuation Effects on lipid profile (total cholesterol, LDL cholesterol, HDL cholesterol, and triglycerides) Effects on metabolic indexes (glucose levels) Effects on metabolic indexes (insulin plasma levels and insulin sensitivity index (HOMA index)) Effects on platelet aggregation Effects on coronary flow reserve	Reduced LDL cholesterol below 130 mg/dL in 56.5% of patients (Combination A) Reduced plasma levels of triglycerides, total and LDL cholesterol, and increased HDL cholesterol Total and LDL cholesterol reduction (Combination B). Reduced plasma levels of glycated hemoglobin, fasting glucose, and insulin, as well as the HOMA index (Combination B) [14]
Study of berberine hydrochloride in the prevention of colorectal adenomas recurrence	Colorectal adenoma	Phase 2	1108 adults sged 18–75 who had at least one, and no more than 6, histologically confirmed colorectal adenomas that were removed within 6 months before recruitment, whose adenoma was not completely removed during a previous colonoscopy; a history of familial adenomatous polyposis or hereditary non-polyposis colorectal cancer (HNPCC, Lynch syndrome); or a history of subtotal/total gastrectomy or partial bowel resection	3 years	Berberine hydrochloride versus placebo	Recurrence rates of colorectal adenoma The incidence of all polypoid lesions or advanced colorectal adenoma or colorectal cancer The incidence of all polypoid lesions or advanced colorectal adenoma or colorectal cancer Changes in fecal microflora	Berberine 0,3 g twice daily was effective and safe The risk of the recurrence of colorectal adenoma was reduced [15]
Berberine effects on clinical symptoms and metabolic disturbance in patients with schizophrenia	Schizophrenia	Phase 4	65 adults aged 18 to 65 years who met the diagnosis of schizophrenia according to the DSM-IV, and have undergone monotherapy of atypical antipsychotics for 4 weeks or more, with at least 60 for positive and negative syndrome scale	8 weeks	Berberine plus any atypical antipsychotic drug as the basic treatment	Positive and Negative Syndrome Scale Changes in Insulin Changes in TC Changes in TG Changes in HDL-C Safety of berberine Changes in CRP Changes in IL-1β Changes in IL-6 Changes in TNF-α	Lowering the concentration of TC and LDL-C in the plasma Significant decrease in fasting insulin and homeostasis model assessment-insulin resistance BMI and serum PRL concentrations have an influence on the improvement of fasting insulin, and homeostasis model assessment-insulin resistance in the berberine group, but not in the placebo group The patient’s response to berberine is BMI-dependent Patients with higher serum prolactin levels had a weaker effect of berberine treatment [16]
Berberine treat metabolic syndrome in schizophrenia	Metabolic syndrome Schizophrenia	Not Applicable	Adult females aged 18–60 with a diagnosis of schizophrenia, undergoing monotherapy of atypical antipsychotics for 2 weeks or more, including olanzapine, clozapine, risperidone, and perphenazine, with a diagnosed metabolic syndrome depending on the guidelines for the prevention and treatment of dyslipidemia in Chinese adults in 2007	8 weeks	Berberine in adjunctive group	Serum fasting blood glucose Serum triglyceride and serum low-density lipoprotein Systolic blood pressure Waistline circumference Diastolic blood pressure	Control of weight gain and other metabolic symptoms associated with antipsychotic therapy as an adjuvant Significant differences in body weight, BMI, and leptin Significant positive correlations with changes in body weight There was no significant difference in adverse events between the two groups [17]
Berberine hyperglycemic clamp	Diabetes mellitus	Phase 1	15 adult healthy males, aged 18–45 with BMI 18–25 kg/m^2^ and a normal oral glucose tolerance test prior to the study, with no family history of diabetes mellitus, and with no medication treatment within 4 weeks prior to the baseline visit, as well as during the study	2 weeks	Berberine versus placebo	Differences in serum insulin levels Differences in serum C-peptide levels Differences in glucose infusion rates Differences in blood glucose levels Heart rate and QT-interval duration	Increase in glucose-dependent insulin secretion Berberine had no effect on insulin secretion at low glucose levels At high glucose levels, insulin release was stimulated by berberine in a dose-dependent manner. Blockade of KCNH6 channels by berberine increased insulin secretion in a glucose-dependent or hyperglycemic manner Berberine did not cause hypoglycemia [18]
Efficacy and safety of berberine in the treatment of diabetes with dyslipidemia	Type 2 diabetes mellitus Metabolic syndrome	Phase 3	120 adults aged 25–70 with newly diagnosed type 2 diabetes, according to the 1999 World Health Organization criteria, with dyslipidemia with a TG of > 150 mg/dL (1.70 mmol/L), and/or TC > 200 mg/dL (5.16 mmol/L), and/or LDL-C > 100 mg/dL (2.58 mmol/L), according to the National Cholesterol Education Program’s Adult Treatment Panel III (NCEP: ATPIII) without previous treatment and with BMI 19–40 kg/m^2^	3 months	Berberine versus placebo	Fasting glucose levels OGTT 2 h glucose levels HbA1c Serum triglycerides Serum total cholesterol HDL-C LDL-C Glucose disposal rate BMI Blood pressure	After 3 months of treatment, plasma glucose levels in the front and under loads were significantly reduced [17]
Therapeutic effects of berberine in patients with type 2 diabetes	Type 2 diabetes	Phase 1 Phase 2	70 adults aged 25 to 75 with a clinical diagnosis of type 2 diabetes with HbA1c > 7.0% or FBG > 7.0 mmol/L with stable or worsening glycemic control for at least 3 months	13 weeks	Berberine versus metformin	HbA1c Blood glucose Blood lipids	Identical effect in the regulation of glucose metabolism (HbA1c, FBG, PBG, fasting insulin, and postprandial insulin), such as metformin. Better regulation of lipid metabolism than metformin-triglycerides, and total cholesterol was significantly lower than in the metformin group Significantly decreased HbA1c levels (HbA1c was comparable to that of metformin) [19]

**Table 2:** Active clinical trials with berberine on the metabolic syndrome and its corresponding diseases based on clinicaltrials.gov (accessed on 10 Fabruary 2022).

Title	Conditions	Status	Phase	Measures
A Research of berberine hydrochloride to prevent colorectal adenomas in patients with previous colorectal cancer	Colorectal adenomas	Recruiting	Phase 2 Phase 3	Cumulative colorectal adenoma Cumulative numbers or diameters of new colorectal adenomas
Comparison of berberine and metformin for the treatment of MS in schizophrenia patients	Schizophrenia Metabolic syndrome	Recruiting	Phase 4	Fasting blood glucose Triglyceride High-density lipoprotein Waist circumference Blood pressure including systolic and diastolic pressure Body mass index Total cholesterol CRP Interleukin-1, interleukin-6, TNF-α
Effect of berberine versus metformin on glycemic control, insulin sensitivity, and insulin secretion in prediabetes	Prediabetes impaired Fasting glucose impaired Glucose tolerance	Active, not recruiting	Phase 4	Fasting glucose levels Postprandial glucose levels Glycosylated hemoglobin Total insulin secretion First phase of insulin secretion Insulin sensitivity Body weight Body mass index Body fat percentage Waist circumference
Effect of berberine for endothelial function and intestinal microflora in patients with coronary artery disease	Stable coronaryartery diseasePercutaneous coronary intervention	Active, not recruiting	Phase 1 Phase 2	Endothelial function Gut microbiome Fecal metabolomics profile Blood lipid levels Inflammatory factor levels Blood glucose levels
Berberine prevents contrast-induced nephropathy in patients with diabetes	Diabetes mellitus Chronic kidney disease	Recruiting	Phase 4	Contrast-induced nephropathy Major adverse renal events
Berberine as adjuvant treatment for schizophrenia patients	Schizophrenia Schizophrenia spectrum and other psychotic disorders Metabolic syndrome X	Recruiting	Phase 2 Phase 3	Weight gain Body mass index Waist circumference Blood pressure Triglycerides Total cholesterol High-density lipoprotein Low-density lipoprotein Fasting glucose Insulin
Evaluating the tolerability and effects of berberine on major metabolic biomarkers: a pilot study	Metabolic syndrome	Recruiting	Not Applicable	LDL cholesterol Hemoglobin A1c Adverse events
Efficacy and safety of berberine in non-alcoholic steatohepatitis	Non-alcoholic steatohepatitis	Recruiting	Phase 4	Histologic features of non-alcoholic steatohepatitis Improvement in the composites of NAFLD activity scores for steatosis, lobular inflammation, and hepatocellular ballooning Improvement in liver histological fibrosis staging Anthropometric measures Blood biochemistry Liver fat content Serum cytokeratin 18
Study on the efficacy and gut microbiota of berberine and probiotics in patients with newly diagnosed type 2 diabetes	Type 2 diabetes	Active, not recruiting	Phase 3	HbA1c Gut microbiome Fasting glucose levels 2-h postprandial glucose levels Fasting insulin levels 2-h postprandial insulin levels Serum triglycerides Serum total cholesterol Serum HDL-C
Effect of berberine hydrochloride on blood pressure and vascular endothelial function in patients With hypertension	Hypertension Endothelial dysfunction Blood pressure	Recruting	Phase 4	Blood pressure Brachial ankle pulse wave velocity Blood pressure Brachial arterial flow-mediated dilation
Effect of berberine on metabolic syndrome, efficacy and safety in combination with antiretroviral therapy in PLWH	Metabolic syndrome HIV-1-infection Glucose intolerance	Not yet recruiting	Phase 3	Insulin resistance Measurement of total cholesterol, HDL, LDL, triglycerides Weight gain or loss measured by kilograms Level of pro-inflammatory cytokines
Berberine prevents contrast-induced nephropathy in patients with diabetes	Diabetes mellitus Chronic kidney disease	Recruiting	Phase 4	Contrast-induced nephropathy Increase in serum creatinine Major adverse renal events
Evaluating the tolerability and effects of berberine on major metabolic biomarkers: a pilot study	Metabolic syndrome	Recruiting	Not Applicable	Change in LDL Cholesterol Change in Hemoglobin A1c Adverse events
Efficacy and safety of berberine in non-alcoholic steatohepatitis	Non-alcoholic steatohepatitis	Recruiting	Phase 4	Histologic features of non-alcoholic steatohepatitis Liver histological fibrosis staging BMI Blood biochemistry Liver fat content Serum cytokeratin 18
Study to determine the effect of synbiotics in patients with pre-diabetes	Pre-diabetes	Recruiting	Not Applicable	Blood glucose levels Metabolic impact of metabolic rheostat and butyrate ultra Gut hormones/incretins (including insulin, C-peptide, glucagon, CCK, GLP-1, GIP, and PYY) Impact of metabolic rheostat and butyrate ultra on food addiction and cravings

## Data Availability

Not applicable.
